# Use of faecal volatile organic compound analysis for ante-mortem discrimination between CWD-positive, -negative exposed, and -known negative white-tailed deer (*Odocoileus virginianus*)

**DOI:** 10.1080/19336896.2019.1607462

**Published:** 2019-04-28

**Authors:** Christine K. Ellis, Steven F. Volker, Doreen L. Griffin, Kurt C. VerCauteren, Tracy A. Nichols

**Affiliations:** a Feral Swine Project, USDA-APHIS-WS-National Wildlife Research Center, Fort Collins, CO, USA; b Analytical Chemistry Department, USDA-APHIS-WS-National Wildlife Research Center, Fort Collins, CO, USA; c BioLaboratories, USDA-APHIS-WS-National Wildlife Research Center, Fort Collins, CO, USA; d USDA-APHIS-VS-Cervid Health Program, Fort Collins, CO, USA

**Keywords:** Chronic wasting disease, CWD, prion, ante-mortem testing, fecal volatile organic compound, VOC

## Abstract

Chronic wasting disease (CWD) is a naturally occurring infectious, fatal, transmissible spongiform encephalopathy of cervids. Currently, disease confirmation relies on post-mortem detection of infectious prions in the medial retropharyngeal lymph nodes or obex in the brain via immunohistochemistry (IHC). Detection of CWD in living animals using this method is impractical, and IHC and other experimental assays are not reliable in detecting low concentrations of prion present in biofluids or faeces. Here, we evaluate the capability of faecal volatile organic compound analysis to discriminate between CWD-positive and -exposed white-tailed deer located at two positive cervid farms, and two groups of CWD-negative deer from two separate disease-free farms.

## Introduction

Chronic wasting disease (CWD) is a naturally occurring, fatal, highly transmissible spongiform encephalopathy [1,2] occurring in susceptible wild, captive, and farmed cervid species (e.g., white-tailed deer (WTD) and mule deer (*Odocoileus virginianus* and *O. hemionus*); North American elk (*Cervus elaphus elaphus*); moose (*Alces alces*); and reindeer (*Rangifer tarandus*) in the United States and Canada [1–8]. Internationally, CWD has been documented in farmed elk in Korea following importation from Canada [2,9], and in wild reindeer and moose in Norway [8,10]. The etiological agent, a prion, an abnormal isoform (PrP^CWD^) of a normal endogenous host prion protein (PrP^c^) [1,2,11], is found in the central nervous system; peripheral nervous system and eyes; and non-neural tissues (e.g., lymphatic system; gastrointestinal, urinary, and reproductive tracts; cardiac and skeletal muscle; glandular tissue; nasal epithelium; and antler velvet) [2,5,12–19]. Infectious prions have also been detected in low concentrations in faeces and biofluids (e.g., blood, milk, saliva, and urine) [3,20–25].

As CWD continues to spread in both captive and wild ungulate populations, detection of infected animals is of great importance. The current ‘gold standard’ diagnostic assay is post-mortem immunohistochemistry (IHC) of the medial retropharyngeal lymph nodes (MRPLN) and obex in the brain. There is a significant need for live animal (i.e., ante-mortem) assays to identify infected animals for disease management and control purposes. Ante-mortem detection of CWD by conventional means has been challenging as IHC cannot be used to test easy to collect samples such as biofluids or faeces [11] and the low concentration of CWD prion present in such samples falls below the detection limit of Western blot. Biopsy and IHC of lymphoid tissue in the recto-anal mucosa (i.e., RAMALT; rectal biopsy) has been shown to have utility under some conditions; however, it is invasive and has limited sensitivity [26] related to the quality of the sample (i.e., too few or no diagnostic lymphoid follicles), repeated sampling of the tissue, extent of histological lesions, and age of the animal [6,27,28].

The development of prion amplification assays such as serial protein misfolding cyclic amplification (PMCA) [24,29–31] and real-time PrPc [32] quaking-induced conversion assay (RT-QuIC) [21,31] allows for amplification of minute, previously undetectable concentrations of infectious prions or their markers to levels detectable in samples such as faeces, urine, saliva, and blood [4,24,25,33,34] although the sensitivity of detection varies between sample type. Additionally, RT-QuIC has demonstrated promise for increased CWD detection in MRPLN and RAMALT samples [35,36]. Like all diagnostic assays, PMCA and RT-QuIC have aspects that make their implementation challenging, such as the level of technical expertise required for reliable results, and the generation of high-quality assay substrate [25,37].

Detection of CWD, particularly in ante-mortem samples, is significantly impacted by genetic variability in the prion protein gene [11,38]. Codon 96 in WTD influences the propagation of the infectious isoform, with wild-type glycine/glycine animals having the shortest incubation period followed by glycine/serine (GS) and serine/serine (SS) individuals. Detection of CWD can be challenging in GS and SS animals; therefore, it is necessary to evaluate the sensitivity of ante-mortem assays in all three genotypes.

Breath and faecal volatile organic compound (VOC) analyses have been explored as non-invasive methods of disease detection. VOCs are organic chemicals that enter a gaseous phase at low temperature, and are produced anthropogenically and biologically by all plant, animals, and microbes. Animals (including humans) produce VOCs via dietary and metabolic pathways, in response to immunologic or inflammatory stimulation, and via host–pathogen interactions. Such VOCs are present in biofluids, breath, and faeces. In humans, a validated breath VOC assay is used to detect *Helicobacter pylori* infection (60–61), and breath, biofluids, and faecal analyses are being explored for diagnosis of metabolic, neoplastic, and infectious disease; dementia; and organ transplant success [39–43]. In domestic and wild ruminants (e.g., cattle (*Bos taurus*), goats (*Capra aegagrus hircus*), WTD, bison (*Bison bison*)), breath and faecal VOC analyses have been used to detect ketosis; bovine tuberculosis; brucellosis; bovine respiratory disease; and Johne’s disease [44–50]. In other species, VOC analysis of serum has been explored for detection of bovine tuberculosis [51].

Development of a method to detect a suite of CWD-specific faecal VOCs would be valuable as a means to detect this disease ante-mortem. Access to portable gas chromatography/mass spectrometry (GCMS) or development of lateral flow assays could feasibly allow on-site testing, and offer lowered cost, increased labour efficiency, and test repeatability. Previously, we have demonstrated that breath and faecal VOCs can be used to discriminate between healthy cattle and cattle experimentally infected with virulent *Mycobacterium bovis* [44]; healthy non-vaccinated and *M. bovis* Bacille-Calumet Guerin (BCG)-vaccinated WTD [50] and cattle [49] prior to and after virulent *M. bovis* challenge; and healthy versus *Brucella abortus*-infected bison [48]. In this study, we explore VOC analysis of faeces as a means to discriminate between CWD-infected, -exposed, and -negative WTD.

## Results

**Figure 1.**
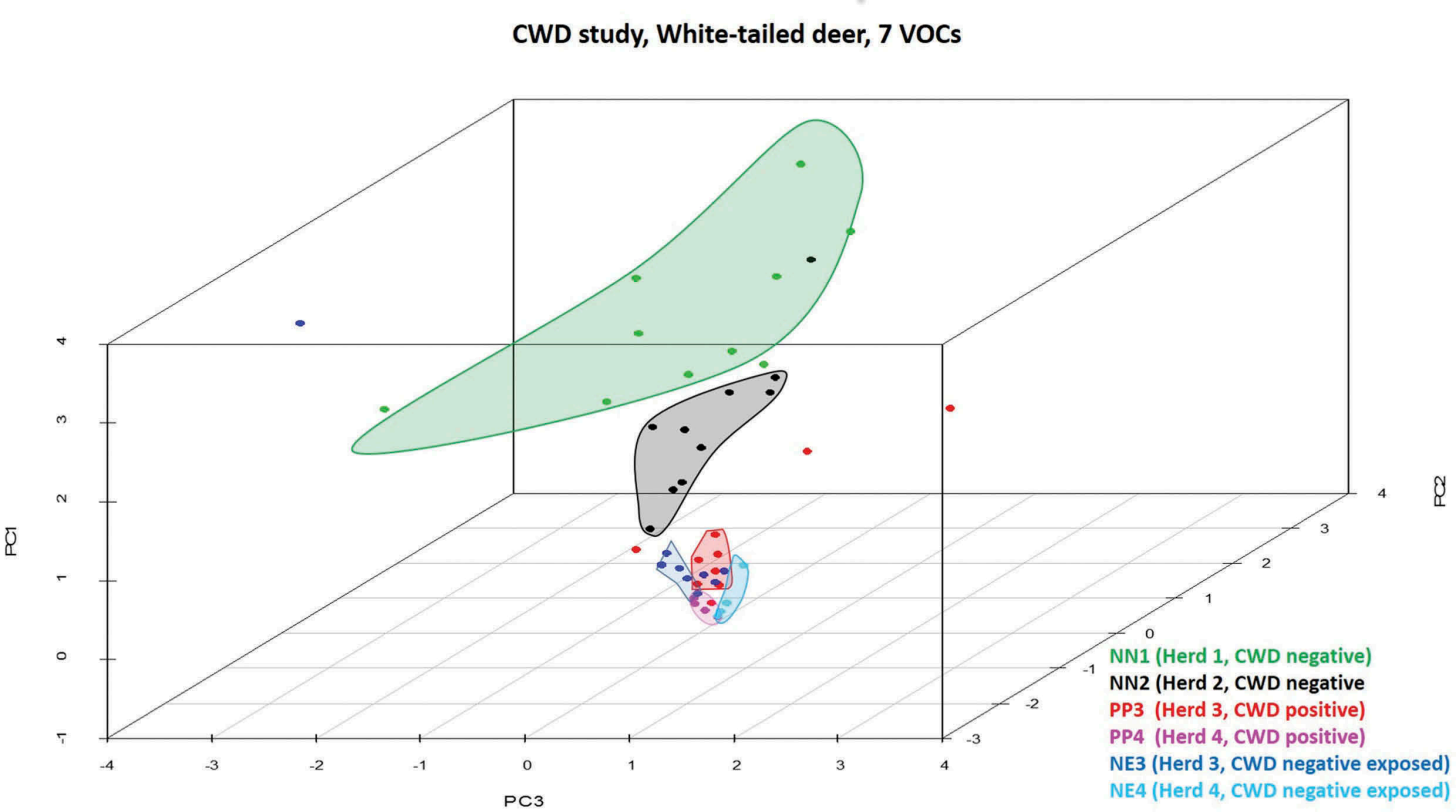
Three-dimensional PCA scatterplot of CWD-positive, -negative exposed, and -negative deer. All known negative animals from Herd 1 (NN1; green dots) and one from Herd 2 (NN2, black dot) are located in a cluster (green) closely associated with all other NN2 individuals (black cluster). Three Herd 3 CWD-positive (PP3, red dots) and one -negative exposed individual (NE3, blue dot) are not associated with clusters in the plot and represent outliers. Remaining PP3 and NE3 animals and Herd 4 (PP4, pink dots; NE4, aqua dots) animals form closely associated clusters, with the exception of four NE3 animals found within or in close association to the PP3 cluster.

**Figure 2.**
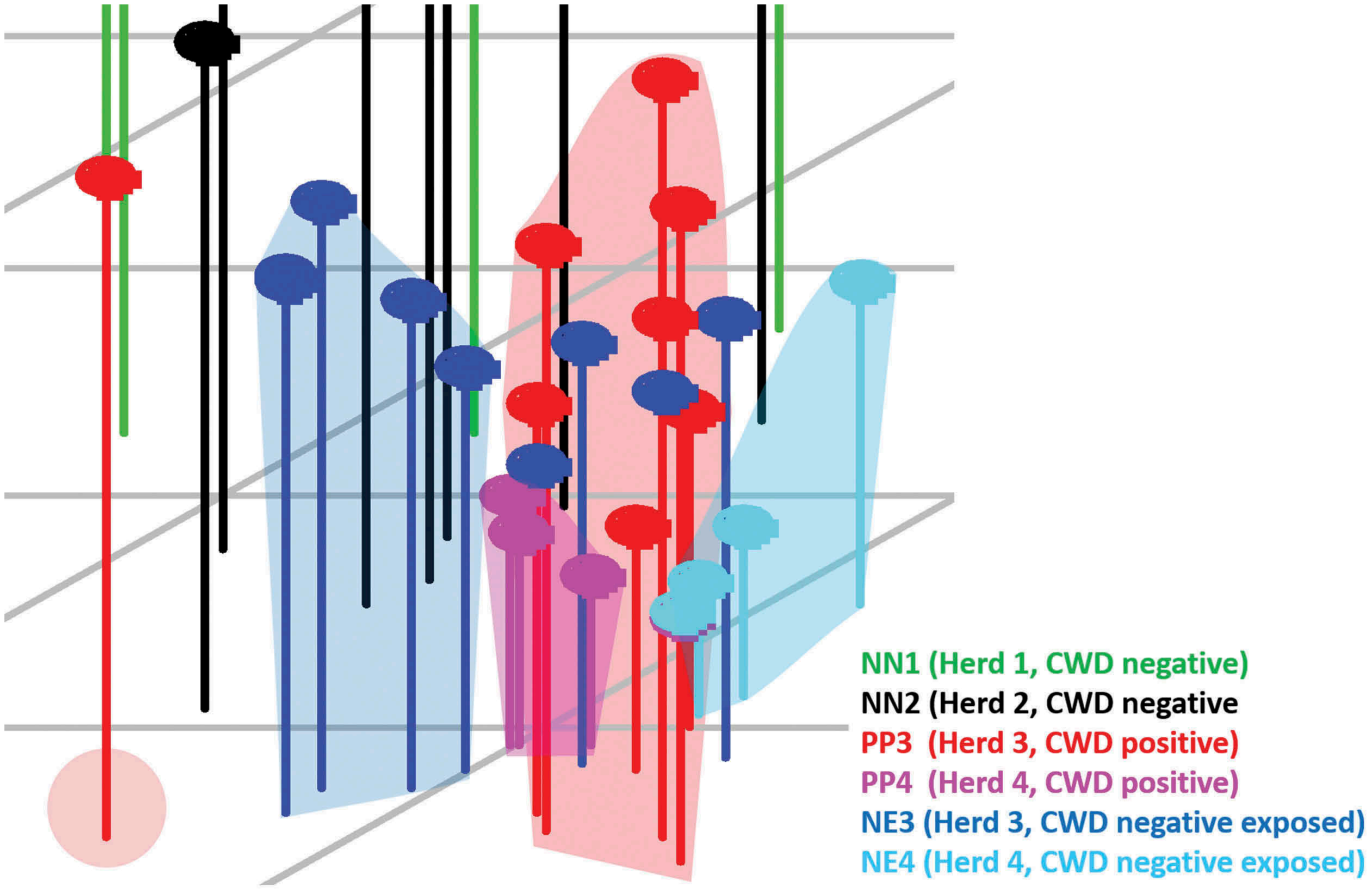
Enlarged view of the area in the PCA scatterplot area containing Herd 3 and 4 CWD-positive and -negative exposed animals. CWD-positive animals from Herd 3 (PP3, red dots) form a distinct cluster containing three negative exposed animals (NE3) from that herd. Remaining NE3 animals form a distinct cluster with the exception of one individual found adjacent to the PP3 cluster. Herd 4 CWD-positive (PP4; pink dots) and -negative exposed (NE4; aqua dots) animals form separate clusters. The close approximation of these clusters indicates that there are some distinct similarities between the cohorts, yet differences between the groups do exist. The three NE3 animals located within the PP3 cluster, and the one NE3 individual located near that cluster may represent animals that were incorrectly classified by our analysis, or may be positive animals infected with a prion burden so low that prion was not detected in the post-mortem IHC analysis performed on the submitted tissues.

The XCMS Online analysis identified 1994 statistically significant ions from a pool of 5265 total ions. Statistically significant ions were GC column retention time matched to 183 total ion chromatographic (TIC) peaks. After excluding peaks potentially associated with diet (*n* = 23) or failing to meet the selection criteria (*n* = 153), a suite of seven candidate peaks remained for statistical analysis. Results of the principal component analysis (PCA) classification performed using all six treatment groups (Figure 1) identified nine animals from confirmed negative Herd 1 (NN1) and four animals from confirmed negative Herd 2 (NN2) within one cluster closely approximated to a second cluster containing five NN2 individuals. One NN1 individual, one CWD-negative exposed individual from Herd 3 (confirmed positive farm; NE3) and two confirmed CWD-positive individuals from Herd 3 (PP3) are located randomly in the plot and represent outliers. Remaining NE3, PP3, one NN2, and all Herd 4 CWD-positive (PP4), and negative exposed (NE4) animals are closely associated but form distinct clusters, which is more readily observed when this area of the scatterplot is enlarged and drop-lines are added to better demonstrate the location of the animals within three-dimensional space (Figure 2). In the enlarged figure (Figure 2), one NN2 individual can be visualized in three-dimensional space between other NN2 individuals and the enlarged clusters of NE and PP animals. One PP3 individual is distinct from all other known positive and negative exposed animals. Remaining PP3 and four NE3 animals form a cluster. Remaining NE3 animals (*n* = 4) form a separate distinct cluster. Three confirmed positive animals from Herd 4 (PP4) form a distinct cluster. The remaining PP4 individual is located in the cluster containing all NE4 animals. Genetics at codon 96 did not appear to influence the sample distribution, as samples fell into their disease status group regardless of their genotype (data not shown). Six class linear discriminant analysis (LDA) classification models were developed using four through seven principal components (PCs) based on the individual and accumulated proportional values of each PC score (Table 1). The optimal model constructed using seven PC scores (100% of data) returned the lowest misclassification rate for the combined data (8%); group misclassification model (Positives = 7%, Negative Exposed = 18%, Known Negatives = 0%); and individual cohort assessment model (PP3 = 10%; PP4 = 0%, NE3 = 10%, NE4 = 20%, NN1 = 0%, NN2 = 0%). In all models, no PP individuals were misclassified as NN animals; misclassifications consisted entirely of NE animals. Negative exposed animals were misclassified in the optimal model as NN, whereas in the models constructed with four or five PCs they were classified as either PP or NN (no NE misclassifications occurred in the model constructed with six PCs). Negative individuals were correctly classified in the optimal model, but were misclassified as PP in the models constructed with four through six PCs. When NE animals are grouped with NN individuals, calculated SN:SP for the classification models constructed with four to seven PCs are 86%:89%; 93%:89%; 93%:89%; and 93%:95%, respectively. When NE animals are grouped with the PP animals, calculated SN:SP are 93%:90%; 97%90%; 97%:90%; and 97%:100%, respectively. The seven VOCs were tentatively identified as an acetal (6,6-dimethoxy-2,5,5-trimethyl-2-hexene); an aldehyde (1-butanol); an alcohol (4-methyl-1-pentanol; isohexanol); an indole (3-methyl 1H indole; skatole); a medium chain fatty acid (hexanoic acid; caproic acid); a phenol (p-cresol; 4-methyl phenol); and a phenylpropane (2-propyl phenol) (Table 2).

**Table 1.** Linear Discriminant Analysis Classification Models of CWD positive and negative deer.

Number of principal components	4	5	6	7
Total misclassification rate (all data combined) (%)	16	12	12	8
Total correct classification rate (all data combined) (%)	84	88	88	92
**Misclassification rate (%) by cohort**				
●PP3 misclassified as PP4	0	0	0	0
●PP3 misclassified as NE3	10	0	10	10
●PP3 misclassified as NE4	0	0	0	0
●PP3 misclassified as NN1	0	0	0	0
●PP3 misclassified as NN2	0	0	0	0
●PP4 misclassified as PP3	0	0	0	0
●PP4 misclassified as NE3	0	0	0	0
●PP4 misclassified as NE4	25	25	0	0
●PP4 misclassified as NN1	0	0	0	0
●PP4 misclassified as NN2	0	0	0	0
●NE3 misclassified as PP3	10	10	0	0
●NE3 misclassified as PP4	0	0	0	0
●NE3 misclassified as NE4	0	0	0	0
●NE3 misclassified as NN1	20	10	10	10
●NE3 misclassified as NN2	0	0	0	0
●NE4 misclassified as PP3	0	0	0	0
●NE4 misclassified as PP4	14	14	29	20
●NE4 misclassified as NE3	0	0	0	0
●NE4 misclassified as NN1	0	0	0	0
●NE4 misclassified as NN2	0	0	0	0
●NN1 misclassified as PP3	0	0	0	0
●NN1 misclassified as PP4	0	0	0	0
●NN1 misclassified as NE3	0	0	0	0
●NN1 misclassified as NE4	0	0	0	0
●NN1 misclassified as NN2	0	0	0	0
●NN2 misclassified as PP3	0	0	0	0
●NN2 misclassified as PP4	20	20	20	0
●NN2 misclassified as NE3	0	0	0	0
●NN2 misclassified as NE4	0	0	0	0
●NN2 misclassified as NN2	0	0	0	0
**Misclassification by group (%)**				
●(PP3 + PP4) misclassified as (NE3 + NE4)	14	7	7	7
●(PP3 + PP4) misclassified as (NN1 + NN2)	0	0	0	0
●(NE3 + NE4) misclassified as (PP3 + PP4)	12	12	12	12
●(NE3 + NE4) misclassified as (NN1 + NN2)	12	6	6	6
●(NN1 + NN2) misclassified as (PP3 + PP4)	10	10	10	0
●(NN1 + NN2) misclassified as (NE3 + NE4)	0	0	0	0
**Correct classification by group (%)**				
●Positive (PP3 + PP4)	86	93	93	93
●Negative exposed (NE3 + NE4)	76	82	82	82
●Negative (NN1 + NN2)	90	90	90	100
Sensitivity when NE3 + NE4 are included with NN1 + NN2	86	93	93	93
Specificity when NE3 + NE4 are included with NN1 + NN2	89	89	89	95
Sensitivity when NE3 + NE4 are included with PP3 + PP4	93	97	97	97
Specificity when NE3 + NE4 are included with PP3 + PP4	90	90	90	100

Eight per cent of all animals in the study were misclassified in the LDA classification model constructed using seven principal component (PC) scores. By cohort, only CWD-positive deer from Herd 3 (PP3), and negative exposed deer from Herds 3 and 4 (NE3, NE4) were misclassified (10%, 10%, 33%, respectively). By group, positive (PP3, PP4) animals were misclassified as NE (7%), and NE individuals were misclassified as PP or known negative (NN) (12% and 6%, respectively). No NN animals were misclassified in the optimal model. Calculated SN:SP when NE3 and NE4 animals were classed as NN were 86%:89%; 93%:89%; 93%:89%; and 93%:95%, respectively. When NE3 and NE4 animals were classified as PP, calculated SN:SP are 100%:90%; 100%90%; 100%:90%; and 100%:100%, respectively.

**Table 2.** Tentative identification of seven peaks used to discriminate between CWD-positive, -negative exposed, and -negative deer.

Peak	Retention time	Tentative identification	Compound family
1	12.532	1-Butanol	Aldehyde
2	13.059	4-Methyl 1-pentanol (isohexanol)	Alcohol
3	17.376	6,6-Dimethoxy-2,5,5-trimethyl 2-hexene	Acetal
4	28.343	Hexanoic acid (caproic acid)	Fatty acid
5	28.535	p-Cresol (4-methyl phenol)	Phenol
6	31.403	2-Propyl phenol	Phenylpropane
7	34.737	3-Methyl 1H indole (skatole)	Indole

Compounds were tentatively identified using a ≥ 65% probability match with AMDIS and NIST software.

Potential metabolic, physiological, or nutritional sources for each tentatively identified compound are summarized in Table 3. Because peer-reviewed literature discussing the metabolism, rumen microbiome, and/or faecal VOCs associated with WTD is not readily available, published information relative to cattle and small domestic ruminants was referred to when necessary. Briefly, 1-butanol is present in some food sources, and is produced endogenously via rumen fermentation [52–55] and the butanoate metabolic pathway [56]. This molecule has been proposed as a biomarker for oxidative damage to lipids, proteins, and DNA [53]. Isohexanol (4-methyl 1-pentanol) is present in all eukaryotes, functions in lipid metabolism and pregnenolone biosynthesis as a precursor to steroid hormone metabolism [57], and has been detected in cattle rumen gas [52]. The third compound (6,6-dimethoxy-2,5,5-trimethyl 2-hexene) is a nutrient utilized for cell signalling, membrane stabilization, and an energy source [53]. Hexanoic acid (caproic acid) is found in some plants, is a rumen fermentation by-product [58,59], and is utilized for cell signalling and multiple metabolic processes [53]. The fifth compound (p-cresol; 4-methyl phenol) is produced by rumen and gastrointestinal microbes [60,61], and is associated with multiple microbial and host metabolic pathways [53,56]. 2-Propylphenol is present in some food sources, and has been isolated from ox urine [62] and the black belly spot of male Iberian red deer (*Cervus elaphus hispanicus*) during rut [63]. Skatole (3-methyl 1H-indole) occurs naturally in faeces [64], is produced by ruminant and monogastric microbes as a by-product of tryptophan degradation [65], and is a principle rumen fermentation metabolite [61].

**Table 3.** Potential sources of tentatively identified volatile organic compounds used to discriminate between CWD positive and negative deer.

Tentative identification	Biological location	Biological function	Ruminant source	Disease association	Miscellaneous
1-Butanol	Mitochondria Muscle tissue	Butanoate metabolism [53,56]	Endogenous Food [53] Microbial metabolism [54] Rumen fermentation [55]	Crohn’s disease (CD) [53] Ulcerative colitis (UC) [53]	Lipids, proteins and DNA oxidative damage biomarker [53] Inhibits intercellular gap-junction communication [66]
Isohexanol (4-methyl 1-pentanol)		Lipid metabolism [53,56] Steroid hormone metabolism [53] Pregnenolone biosynthesis [57]	Rumen fermentation [52]		Exists in all eukaryotes [53]
6,6-Dimethoxy-2,5,5-trimethyl 2-hexene	Cell membrane	Cell signalling [53] Membrane stabilizer [53] Energy source and storage [53] Nutrient [53]	Endogenous [53] Food [53]		
Hexanoic acid (caproic acid)	Cytoplasm Cell membrane Adiposome	Cell signalling, lipid transport, metabolism, and peroxidation. Β-oxidation of very long chain fatty acids and mitochondrial β-oxidation of short chain fatty acids [53] Energy source and storage [53] Fatty acid biosynthesis [53,67]	Endogenous [53] Food [53] Rumen fermentation [52,58,68] Detected in cattle breath [59]		Found in animal fats and oils; some plants. Abnormal concentrations in humans with autism, pervasive developmental disorder, CD, UC, Immunoglobulin A nephropathy [53]
p-Cresol (4-methyl phenol)	Adipose tissue Cell membrane Fibroblasts	Protein, tyrosine, toluene, phenylalanine metabolism [53,67]	Rumen and intestinal bacteria metabolism of protein [53,60,69]. Present in cattle faeces [49] Present in WTD faeces [50]	Inhibits phagocyte function; decreases leukocyte adhesion to cytokine-stimulated endothelial cells [53] Decreased in *M. bovis* infection [49,50]	Diminishes oxygen uptake in rat brain. Alters bacterial cell membrane permeability. Blocks cell potassium channels [53]
4-Propyl phenol	Cell membrane		Endogenous [53] Food [53] VOC in ox urine [62] VOC associated Iberian red deer [63]		
Skatole (3-methyl-1H indole)	Cell membrane	Tryptophan metabolism [53,67] Bacterial intercellular signal molecule [70] Some derivatives function as neurotransmitters [71]	Endogenous [53] Food [53] Rumen, gastrointestinal metabolite [52] Present in cattle faeces [49] Present in WTD faeces [50]	Abnormal concentration in humans with autism, UC, CD [53] Increased in *M. bovis* infection [49,50]	

Most tentatively identified VOCs serve as endogenous or exogenous nutrients, metabolites or are by-products of microbial or host metabolism. Several compounds (1-butanol, hexanoic acid, p-cresol, skatole) appear related to disease presence or immune system function.

## Discussion

The ‘holy grail’ of VOC analysis for disease detection, regardless of the sample used, is identification of a disease-specific biomarker. This has happened occasionally, with most successes occurring relative to metabolic diseases [40,66]; however, there has been little success identifying a unique biomarker paired to a specific infectious disease. Because identifying the sources of infectious disease related VOCs is difficult to determine, it has been hypothesized that detected VOCs may represent some ubiquitous metabolic or immunological response to disease. Based upon the literature and metabolomics database searches we conducted, many of the compounds we tentatively identified are related to metabolic function and synthesis of endogenous substances. Some appear to have immunological function, serve as biomarkers for oxidative damage, and occur in altered concentrations relative to the presence of neurological, metabolic, and infectious disease. In previous studies, we have successfully identified suites of VOCs that allowed discrimination between healthy and *M*. *bovis*-infected cattle [44,49] and WTD [50], and healthy vs. *B*. *abortus*-infected American bison (*B*. *bison*) [48]. Such was the case in this study. Interestingly, five compounds used for discrimination between cohorts in this study (1-butanol, 2-propyl phenol, 6,6-dimethoxy-2,5,5-trimethyl 2-hexene, hexanoic acid, isohexanol) were not present in the suites of faecal VOCs we used to differentiate between healthy and *M*. *bovis*-infected cattle and WTD. This finding is suggestive that disease-specific suites of VOCs may exist, and represents an area of study that should be further explored before such a relationship can be claimed.

This manuscript summarizes the first study exploring faecal VOC analysis as a method to discriminate between CWD-positive, -exposed and -negative WTD with inference to use of this modality as an ante-mortem test for CWD surveillance of captive, farmed, and wild ungulate populations. Data were analysed using two statistical methods (e.g., PCA and LDA). The strength of PCA as a statistical tool is its independence in pattern recognition, as the analysis occurs independent of treatment group designation. As such, PCA was utilized first to transform the highly variable chromatographic data into orthogonal linearly uncorrelated data (i.e., PC scores), and to generate a visual representation of the data (i.e., the PCA scatterplot). The scatterplot then visually presents the WTD as grouped by the PCA without regard for any treatment group designation. The LDA was performed using our treatment group designations, with the intent to model the difference or similarity between those groups. Results are identified as animals correctly classified to their respected treatment group or misclassified into another treatment group.

We were able to correctly identify individuals within the three cohorts using LDA classification models with good accuracies. Of special importance are results in all models in which no CWD-positive animals were misclassified as CWD-negative. Misclassification of CWD-negative exposed animals did occur, with animals misclassified as CWD-positive (12%) or CWD-negative (6%). Misclassification of CWD-negative exposed individuals as CWD-positive can be interpreted as beneficial (i.e., it is better to classify animals with a subclinical, highly infectious fatal disease as positive) relative to disease control. These misclassifications could be due to failure of our classification models or it is possible that these individuals might be CWD-positive and were incorrectly classified prior to our study if their infectious prion burden was low, and the post-mortem IHC was performed on MRPLN or obex tissue that did not happen to contain detectable infectious prion.

Efforts to control for genetic, local environmental effects and diet were used when selecting the suite of VOCs used in our final analysis; however, it is likely that some genetic, geographical, environmental, and dietary effects did influence the changes noted in the VOCs used. Because housing large groups of WTD infected with a chronic infectious disease in under tightly controlled environmental and dietary conditions for long periods of time is difficult and expensive, reliance on ‘real-world’ scenarios is often the route required to acquire CWD samples. While this potentially confounds our results, it does represent testing of ‘real-world’ samples, and our results demonstrate robust potential for our analysis method. To further understand the potential of faecal VOC analysis as a means to detect CWD presence or absence, more blinded studies should be undertaken to increase the number of animals, disease status, and geographic localities from which samples are drawn and to increase the database.

While our use of three group diagnostic classification does not compare strictly with standard estimates of SN and SP, some comparison to other assays are possible (Table 4). The published SN:SP for ante-mortem IHC on tonsil or RAMALT biopsies are 99%:100% and 68%:99% [11,67–69], indicating that 1–32% of CWD-positive animals would be incorrectly identified as CWD-negative, while 0–1% of CWD-negative animals would be identified as CWD positive. The SN:SP for the RT-QuIC assay performed on RAMALT is reported as 70%:94% [11,69], indicating that 30% of CWD-infected animals and 6% of disease-free animals would be identified falsely negative and falsely positive, respectively. When RT-QuIC and/or PMCA were performed on nasal brushings, biofluids, or faeces, resulting SN:SP ranges were 16–93%:96–100% [11,69–74] (e.g., 7–84% of CWD-positive individuals tested false negative; 0–4% of CWD-negative animals tested false positive) depending on the assay and sample used. Calculated SN:SP in this study when CWD-negative exposed animals were grouped with CWD-negatives ranged 86–93%:89–95% (e.g., 7–14% of CWD-positive animals and 5–11% of CWD-negative animals incorrectly identified as false negative and false positive, respectively). When CWD-negative exposed animals were included in the CWD-positive group, the SN:SP ranged 93–97%:90–100%. In this calculation, all CWD-positive animals were correctly identified, and 0–10% of CWD-negative individuals were incorrectly identified false positive. A possible explanation for the difference in the results may be genetic, geographical, or dietary effects that could not be controlled for that changed the SN: SP in the second calculation method. An unfortunate limitation to all of these assays is the difficulty in accurately assessing the disease status of negative exposed individuals.

**Table 4.** Comparison of CWD assay sensitivities (SN) and specificities (SP).

		Assay				
Test application	Tissue	IHC	ELISA	PMCA	RT-QuIC^a^	References
Post-mortem	Obex	99:100	92:100			[11,75,76]
Post-mortem	MRLN		99:99		100:100	[11,69,75,76]
Ante-mortem	MRPLN	99:100				[11,67,68]
Ante-mortem	RAMALT	68:99			70:94	[11,69]
Ante-mortem	Nasal Brushing				16:91	[11,69]
Ante-mortem	Blood				93:100	[11,70]
Ante-mortem	CSF			19:100	50:96	[11,71]
Ante-mortem	Saliva				78:98	[11,72]
Ante-mortem	Urine				39:100	[11,72]
Ante-mortem	Faeces			NA:NA	53:100	[11,73,74]

Published results of studies evaluating the capability of various assays to detect CWD prion in a variety of samples are presented. Post-mortem (i.e., gold standard) IHC and ELISA SN: SP are included for comparison. IHC: immunohistochemistry; ELISA: enzyme-linked immunosorbent assay; PAMP: protein misfolding cyclic amplification; RT-QuIC: recombinant PrPc quaking induced conversion assay; MRPLN: medial retropharyngeal lymph node; RAMALT: rectoanal mucosal associated lymphoid tissue; CSF: cerebrospinal fluid. ^a^Potential ante-mortem assay.

An interesting finding in this study was that misclassified CWD-negative exposed animals were identified as CWD-positive or -negative individuals. This finding opens questions of whether VOC analysis is capable of discriminating between subclinically infected and true negative individuals in a positive herd, how this testing strategy might compare to RT-QuIC and PMCA results, and whether the disease status of such individuals might change over time. Unfortunately, following the negative exposed animals over time was not an option for this study, but this could be a potential area to evaluate in the future.

The results of this preliminary study exploring use of faecal VOC analysis as a means to discriminate between CWD-positive, -negative exposed, and -known negative animals are encouraging. The sample size used in this pilot study was small; therefore, additional studies should utilize larger sample sizes in order to test the robustness of this method as a potential diagnostic tool. All of the CWD-positive deer in this study were positive in both the MRPLN and the obex, indicating that the disease was significantly progressed in those individuals. Other ante-mortem diagnostic assays such as PMCA performed on blood samples have been successful in detecting CWD in deer that were positive in both locations as well. However, animals that are CWD-positive only in the MRPLN are in earlier stages of the disease course, and are therefore of the most interest for early CWD detection; however, detection of infectious prion by PMCA and other assays is significantly reduced in animals that are positive only in the MRPLN [34]. Further studies utilizing VOC testing must include animals that are positive in the MRPLN only as well as MRPLN and obex for comparison. Additionally, because CWD has been detected in faecal samples by PMCA and RT-QuIC [73,74], it would be very informative to use faecal VOC analysis in tandem with one or both of these assays for comparison. Should faecal VOC analysis prove robust in discriminating between CWD-positive and -negative animals, and sensitive enough to detect subclinical infection in negative exposed individuals, it would provide a powerful tool for disease detection and management. This assay would also vastly improve the ability of wildlife managers to perform wild cervid CWD surveillance from environmental samples and reduce reliance on hunter-harvested or lethal sampling.

## Materials and methods

### Sample collection

In cooperation with state and federal agencies, faecal samples were opportunistically collected post-mortem from 51 farmed WTD at four different locations (Table 5). Herds 1 and 2 were located at farms confirmed free from CWD. Herds 3 and 4 were located at confirmed CWD-positive farms with prevalence rates of >50% and <20%, respectively. All herds were separated geographically and environmentally from each other, and fed different diets relative to location and owner preference. Animals in the CWD-positive herds were depopulated for disease control purposes, then tested for CWD via IHC of the MRPLN and obex by the United States Department of Agriculture (USDA) National Veterinary Services Laboratory in Ames, Iowa, USA, as previously described [38], and genotyped at codon 96 by GeneCheck^TM^ [75]. All faeces were placed in individual 50 ml conical tubes and stored on ice until transport to the USDA-Animal Plant Health and Inspection Service-Wildlife Service-National Wildlife Research Center where they were stored at −80°C until analysis.

**Table 5.** Herd identification, animal classification, CWD status, and the number of animals from each sample group used in the study.

Herd	Animal classification	CWD status	Number of animals
1	Known Negative (NN1)	Non-Detect	10
2	Known Negative (NN2)	Non-Detect	10
3	Negative Exposed (NE3)	Non-Detect	10
3	Positive (PP3)	Positive	10
4	Negative Exposed (NE4)	Non-Detect	7
4	Positive (PP4)	Positive	4

Known Negative (NN): deer not exposed to CWD. Negative Exposed (NE): deer resided with known CWD-positive cohorts but no prion detected by IHC post-mortem. Positive (PP): deer were identified as CWD-positive by post-mortem immunohistochemistry (IHC) in both the medial retropharyngeal lymph nodes (MRPLN) and the obex.

### GCMS analysis

A 1.5 gm aliquot was removed from each faecal sample and placed into the bottom of a clean 20 ml glass vial (Supelco Part # SU860097, MilliporeSigma, St. Louis, MO, USA) sealed with a screw-top lid containing a polytetrafluorethylene (PTFE)-lined silicone septum (Supelco Part # SU860101, MilliporeSigma, St. Louis, MO, USA) for analysis by GCMS. Each sample was warmed to room temperature and an internal standard (0.010 mL, 70 ppm (+) carvone in water) added prior to randomized placement into a GC 120 PAL autosampler (Agilent Technologies, Santa Clara, CA, USA). Samples were pre-incubated for 10 min at 37°C with pulsed agitation (250 RPM for 5 s, off for 2 s) followed by extraction of vial headspace VOCs using a solid-phase microextraction (SPME) fibre (StableFlex™ 2 cm divinylbenzene/carboxen/polydimethylsiloxane (DVB/CAR/PDMS), Supelco Inc., Bellefonte PA, USA). Extraction time was 40 min at 37°C with pulsed agitation. Following VOCs extraction, the SPME was inserted into the splitless injection port (270°C with 0.75 mm ID, ultra-inert straight liner) equipped with a 23 ga Merlin Microseal™ septa (Merlin Instrument Company, Half Moon Bay, CA, USA) in an Agilent 7890B GC (Agilent Technologies) for desorption for 1 min. A Stabilwax®-DA 30 m × 0.25 mm ID × 0.25 µm film thickness (Restek Corporation, Bellefonte, PA, USA) column was used with helium carrier gas in constant flow mode (1.0 mL/min). The GC oven temperature was held at 35°C for 2.5 min, increased at 6.0°C/min to 260°C, and then held for 5 min. The GC was coupled through a 280°C transfer line with an Agilent 5977A mass selective detector (MSD) equipped with an extractor electron impact source operated at 230°C. The MSD quadrupole was operated at 150°C and the scan range was 50–500 m/z.

### Data analysis

Chromatographic data were analysed as described in Ellis et al. [49]. Briefly, baseline-corrected chromatograms were first evaluated using the XCMS Online multi-group comparison feature [49,77,78] (www.xcmsonline.scripps.edu) to identify statistically significant peak ion abundances which were then retention time matched to TIC peaks present in the each sample chromatogram using Agilent ChemStation software (Agilent Technologies). Peaks exclusive to location of sampling were excluded to remove potential dietary sources of compounds, and an optimal suite of peaks was identified using peak selection criteria (e.g., between groups fold differences ≥3.0; biological relevance). A PCA was performed on the optimal suite of peaks to transform the chromatographic data into orthogonal (linearly uncorrelated) PC scores and to generate a visual scatterplot of the data. The PC scores were then utilized in a LDA to evaluate the capability of the selected suite of peaks to discriminate between the study subjects and correctly identify their CWD status and Herd designation. All statistical analyses were performed using statistical packages available in ‘R’ [79,80]. Sensitivity (SN) and Specificity (SP) were calculated using the following formulas [81,82]:

SN=Truepositives/Truepositives+FalseNegatives

SP=Truenegatives/Truenegatives+FalsePositives

Because the ‘true’ disease status of the negative exposed animals from Herds 3 and 4 (NE3, NE4) could not be confidently assumed, SN:SP calculations were performed first by including them with the Herds 1 and 2 known negative animals (NN1, NN2) as ‘true negatives;’ and second, by including them with known positive animals in Herds 3 and 4 (PP3, PP4) as ‘true positives.’

Automated Mass Spectral Deconvolution and Identification System software [83,84] (www.amdis.net); a standard chemical database (National Institute of Standards and Technology W8N08 (www.nist.gov)); and two metabolomics databases (Kyoto Encyclopedia of Genes and Genomes [56,85] (KEGG; www.genome.jp) and Human Metabolome Database [53] (HMDB; www.hmdb.org) were used to tentatively identify each peak. Peaks meeting minimum spectral match probability ≥65% were further evaluated using KEGG, HMDB, and peer-reviewed literature to determine if the tentatively identified compounds were associated with ruminant physiology or prion-associated disease. Chemical standards were not used to definitively identify selected VOCs due to cost and lack of access to a chemical standards library.

## References

[1] Haley NJ, Hoover EA. Chronic wasting disease of cervids: current knowledge and future perspectives. Annu Rev Anim Biosci. 2015;3(1):305–325. 25387112 10.1146/annurev-animal-022114-111001

[2] Bourne D Chronic wasting disease of cervids. Small Rumin Res. 2015;128:72–78.

[3] Nichols TA, Spraker TR, Gidlewski T, et al Detection of prion protein in the cerebrospinal fluid of elk (Cervus canadensis nelsoni) with chronic wasting disease using protein misfolding cyclic amplification. J Vet Diagn Invest. 2012;24(4):746–749. 22621952 10.1177/1040638712448060

[4] Pulford B, Spraker TR, Wyckoff AC, et al Detection of PrPCWD in feces from naturally exposed Rocky Mountain elk (Cervus elaphus nelsoni) using protein misfolding cyclic amplification. J Wildl Dis. 2012;48(2):425–434. 22493117 10.7589/0090-3558-48.2.425

[5] Spraker T, Zink R, Cummings B, et al Distribution of protease-resistant prion protein and spongiform encephalopathy in free-ranging mule deer (Odocoileus hemionus) with chronic wasting disease. Vet Pathol. 2002;39(5):546–556. 12243464 10.1354/vp.39-5-546

[6] Spraker TR, VerCauteren KC, Gidlewski T, et al Antemortem detection of PrPCWD in preclinical, ranch-raised rocky mountain Elk (Cevvus Elaphus Nelsoni) by Biopsy of the Rectal Mucosa. J Vet Diagn Invest. 2009;21(1):15–24. 19139496 10.1177/104063870902100103

[7] Baeten LA, Powers BE, Jewell JE, et al A natural case of chronic wasting disease in a free-ranging moose (Alces alces shirasi). J Wildl Dis. 2007;43(2):309–314. 17495319 10.7589/0090-3558-43.2.309

[8] Benestad SL, Mitchell G, Simmons M, et al First case of chronic wasting disease in Europe in a Norwegian free-ranging reindeer. Vet Res. 2016;47(1):88. 27641251 10.1186/s13567-016-0375-4 PMC5024462

[9] Tae-Yung K, Hyun-Joo S, Yi-Seok J, et al Additional cases of chronic wasting disease in imported deer in Korea. J Vet Med Sci. 2005;67(8):753–759. 16141661 10.1292/jvms.67.753

[10] Ricci A, Allende A, Bolton D, et al Chronic wasting disease (CWD) in cervids. Efsa J. 2017;15:1. 10.2903/j.efsa.2017.4667 PMC7010154 32625260

[11] Haley NJ, Richt JA Evolution of Diagnostic Tests for Chronic Wasting Disease, a Naturally Occurring Prion Disease of Cervids. Pathogens. 2017;6(3):35. 10.3390/pathogens6030035 PMC5617992 28783058

[12] Sigurdson CJ, Williams ES, Miller MW, et al Oral transmission and early lymphoid tropism of chronic wasting disease PrPres in mule deer fawns (Odocoileus hemionus). J Gen Virol. 1999;80(10):2757–2764. 10573172 10.1099/0022-1317-80-10-2757

[13] Daus ML, Breyer J, Wagenfuehr K, et al Presence and seeding activity of pathological prion protein (PrP TSE) in skeletal muscles of white-tailed deer infected with chronic wasting disease. PLoS One. 2011;6(4):e18345. 21483771 10.1371/journal.pone.0018345 PMC3069970

[14] Jewell JE, Brown J, Kreeger T, et al Prion protein in cardiac muscle of elk (Cervus elaphus nelsoni) and white-tailed deer (Odocoileus virginianus) infected with chronic wasting disease. J Gen Virol. 2006;87(11):3443–3450. 17030881 10.1099/vir.0.81777-0

[15] Mathiason CK, Hayes-Klug J, Hays SA, et al B cells and platelets harbor prion infectivity in the blood of deer infected with chronic wasting disease. J Virol. 2010;84(10):5097–5107. 20219916 10.1128/JVI.02169-09 PMC2863796

[16] Haley NJ, Mathiason CK, Carver S, et al Detection of chronic wasting disease prions in salivary, urinary, and intestinal tissues of deer: potential mechanisms of prion shedding and transmission. J Virol. 2011;85(13):6309–6318. 21525361 10.1128/JVI.00425-11 PMC3126547

[17] Peralta OA, Eyestone WH Quantitative and qualitative analysis of cellular prion protein (PrPC) expression in bovine somatic tissues. Prion. 2009;3(3):161–170. 19806026 10.4161/pri.3.3.9772 PMC2802781

[18] Monello RJ, Powers JG, Hobbs NT, et al Efficacy of antemortem rectal biopsies to diagnose and estimate prevalence of chronic wasting disease in free-ranging cow elk (Cervus elaphus nelsoni). J Wildl Dis. 2013;49(2):270–278. 23568902 10.7589/2011-12-362

[19] Wild MA, Spraker TR, Sigurdson CJ, et al Preclinical diagnosis of chronic wasting disease in captive mule deer (Odocoileus hemionus) and white-tailed deer (Odocoileus virginianus) using tonsillar biopsy. J Gen Virol. 2002;83(10):2629–2634. 12237447 10.1099/0022-1317-83-10-2629

[20] Notari S, Moleres FJ, Hunter SB, et al Multiorgan detection and characterization of protease-resistant prion protein in a case of variant CJD examined in the United States. PLoS One. 2010;5(1):e8765. 20098730 10.1371/journal.pone.0008765 PMC2808239

[21] Selariu A, Powers JG, Nalls A, et al In utero transmission and tissue distribution of chronic wasting disease-associated prions in free-ranging Rocky Mountain elk. J Gen Virol. 2015;96(11):3444–3455. 26358706 10.1099/jgv.0.000281 PMC4806583

[22] Elder AM, Henderson DM, Nalls AV, et al Immediate and ongoing detection of prions in the blood of hamsters and deer following oral, nasal, or blood inoculations. J Virol. 2015;89(14):7421–7424. 25926635 10.1128/JVI.00760-15 PMC4473550

[23] Elder AM Detecting the Temporal Status of Blood-borne Prions in Transmissible Spongiform Encephalopathy-Infected Hosts: Colorado State University Libraries; 2015.

[24] John TR, Schätzl HM, Gilch S Early detection of chronic wasting disease prions in urine of pre-symptomatic deer by real-time quaking-induced conversion assay. Prion. 2013;7(3):253–258. 23764839 10.4161/pri.24430 PMC3783112

[25] Henderson DM, Manca M, Haley NJ, et al Rapid antemortem detection of CWD prions in deer saliva. PLoS One. 2013;8(9):e74377. 24040235 10.1371/journal.pone.0074377 PMC3770611

[26] Thomsen BV, Schneider DA, O’Rourke KI, et al Diagnostic accuracy of rectal mucosa biopsy testing for chronic wasting disease within white-tailed deer (Odocoileus virginianus) herds in North America: effects of age, sex, polymorphism at PRNP codon 96, and disease progression. J Vet Diagn Invest. 2012;24(5):878–887. 22914819 10.1177/1040638712453582

[27] Geremia C, Hoeting JA, Wolfe LL, et al Age and repeated biopsy influence antemortem PRPCWD testing in mule deer (Odocoileus hemionus) in Colorado, USA. J Wildl Dis. 2015;51(4):801–810. 26251986 10.7589/2014-12-284

[28] Fox KA, Jewell JE, Williams ES, et al Patterns of PrPCWD accumulation during the course of chronic wasting disease infection in orally inoculated mule deer (Odocoileus hemionus). J Gen Virol. 2006;87(11):3451–3461. 17030882 10.1099/vir.0.81999-0

[29] Tamgüney G, Miller MW, Wolfe LL, et al Asymptomatic deer excrete infectious prions in faeces. Nature. 2009;461(7263):529. 19741608 10.1038/nature08289 PMC3186440

[30] Johnson CJ, Phillips KE, Schramm PT, et al Prions adhere to soil minerals and remain infectious. PLoS Pathog. 2006;2(4):e32. 16617377 10.1371/journal.ppat.0020032 PMC1435987

[31] Saá P, Cervenakova L Protein misfolding cyclic amplification (PMCA): current status and future directions. Virus Res. 2015;207:47–61. 25445341 10.1016/j.virusres.2014.11.007

[32] Nichols T, Pulford B, Wyckoff AC, et al Detection of protease-resistant cervid prion protein in water from a CWD-endemic area. Prion. 2009;3(3):171–183. 19823039 10.4161/pri.3.3.9819 PMC2802782

[33] Orru CD, Wilham JM, Vascellari S, et al New generation QuIC assays for prion seeding activity. Prion. 2012;6(2):147–152. 22421206 10.4161/pri.19430 PMC7082091

[34] Kramm C, Pritzkow S, Lyon A, et al Detection of prions in blood of cervids at the asymptomatic stage of chronic wasting disease. Sci Rep. 2017;7(1):17241. 29222449 10.1038/s41598-017-17090-x PMC5722867

[35] Haley NJ, Henderson DM, Wycoff S, et al Chronic wasting disease management in ranched elk using rectal biopsy testing. Prion. 2018;12(2):1–16. 10.1080/19336896.2018.1436925 PMC6016512 29424295

[36] Manne S, Kondru N, Nichols T, et al Ante-mortem detection of chronic wasting disease in recto-anal mucosa-associated lymphoid tissues from elk (Cervus elaphus nelsoni) using real-time quaking-induced conversion (RT-QuIC) assay: A blinded collaborative study. Prion. 2017;11(6):415–430. 29098931 10.1080/19336896.2017.1368936 PMC5786361

[37] Rubenstein R, Piltch MS, Gray PC Rapid antemortem detection of infectious agents. Google Patents; 2010.

[38] Nichols TA, Spraker TR, Rigg TD, et al Intranasal inoculation of white-tailed deer (Odocoileus virginianus) with lyophilized chronic wasting disease prion particulate complexed to montmorillonite clay. PLoS One. 2013;8(5):e62455. 23671598 10.1371/journal.pone.0062455 PMC3650006

[39] Garner CE, Smith S, Bardhan P, et al A pilot study of faecal volatile organic compounds in faeces from cholera patients in Bangladesh to determine their utility in disease diagnosis. Trans R Soc Trop Med Hyg. 2009;103(11):1171–1173. 19268999 10.1016/j.trstmh.2009.02.004

[40] Probert CS, Khalid T, Ahmed I, et al Volatile organic compounds as diagnostic biomarkers in gastrointestinal and liver diseases. J Gastrointest Liver Dis. 2009;18(3):337–343. 19795029

[41] Phillips M, Basa-Dalay V, Bothamley G, et al Breath biomarkers of active pulmonary tuberculosis. Tuberculosis. 2010;90(2):145–151. 20189456 10.1016/j.tube.2010.01.003

[42] Phillips M, Gleeson K, Hughes JMB, et al Volatile organic compounds in breath as markers of lung cancer: a cross-sectional study. Lancet. 1999;353(9168):1930–1933. 10371572 10.1016/S0140-6736(98)07552-7

[43] Shirasu M, Touhara K The scent of disease: volatile organic compounds of the human body related to disease and disorder. J Biochem. 2011;150(3):257–266. 21771869 10.1093/jb/mvr090

[44] Ellis CK, Stahl RS, Nol P, et al A pilot study exploring the use of breath analysis to differentiate healthy cattle from cattle experimentally infected with Mycobacterium bovis. PloS one. 2014;9(2):e89280. 24586655 10.1371/journal.pone.0089280 PMC3933422

[45] Burciaga-Robles LO, Holland BP, Step DL, et al Evaluation of breath biomarkers and serum haptoglobin concentration for diagnosis of bovine respiratory disease in heifers newly arrived at a feedlot. Am J Vet Res. 2009;70(10):1291–1298. 19795945 10.2460/ajvr.70.10.1291

[46] Knobloch H, Köhler H, Commander N, et al, editors. Volatile organic compound (VOC) analysis for disease detection: proof of principle for field studies detecting paratuberculosis and brucellosis. OLFACTION AND ELECTRONIC NOSE: Proceedings of the 13th International Symposium on Olfaction and Electronic Nose, Ann Arbor, MI, USA AIP Publishing; 2009.

[47] Purkhart R, Köhler H, Liebler-Tenorio E, et al Chronic intestinal Mycobacteria infection: discrimination via VOC analysis in exhaled breath and headspace of feces using differential ion mobility spectrometry. J Breath Res. 2011;5(2):027103. 21512209 10.1088/1752-7155/5/2/027103

[48] Bayn A, Nol P, Tisch U, et al Detection of volatile organic compounds in brucella abortus-seropositive Bison. Anal Chem. 2013;85(22):11146–11152. 24156543 10.1021/ac403134f

[49] Ellis CK, Rice S, Maurer D, et al Use of fecal volatile organic compound analysis to discriminate between non-vaccinated and BCG—vaccinated cattle prior to and after Mycobacterium bovis challenge. PloS one. 2017;12(7):e0179914. 28686691 10.1371/journal.pone.0179914 PMC5501492

[50] Stahl RS, Ellis CK, Nol P, et al Fecal Volatile Organic Ccompound Profiles from White-Tailed Deer (Odocoileus virginianus) as Indicators of Mycobacterium bovis Exposure or Mycobacterium bovis Bacille Calmette-Guerin (BCG) Vaccination. PloS One. 2015;10(6):e0129740. 26060998 10.1371/journal.pone.0129740 PMC4465024

[51] Fend R, Geddes R, Lesellier S, et al Use of an electronic nose to diagnose Mycobacterium bovis infection in badgers and cattle. J Clin Microb. 2005;43(4):1745–1751. 10.1128/JCM.43.4.1745-1751.2005 PMC1081320 15814995

[52] Cai L, Koziel JA, Davis J, et al Characterization of volatile organic compounds and odors by in-vivo sampling of beef cattle rumen gas, by solid-phase microextraction, and gas chromatography–mass spectrometry–olfactometry. Anal Bioanal Chem. 2006;386(6):1791–1802. 17009001 10.1007/s00216-006-0799-1

[53] Wishart DS, Jewison T, Guo AC, et al HMDB 3.0—the human metabolome database in 2013. Nucleic Acids Res. 2012;41(D1):D801–D807. 23161693 10.1093/nar/gks1065 PMC3531200

[54] Sauer M Industrial production of acetone and butanol by fermentation—100 years later. FEMS Microbiol Lett. 2016;363(13):fnw134. 27199350 10.1093/femsle/fnw134 PMC4894279

[55] Parmar NR, Kumar JN, Joshi CG Deep insights into carbohydrate metabolism in the rumen of Mehsani buffalo at different diet treatments. Genom Data. 2015;6:59–62. 26697334 10.1016/j.gdata.2015.08.007 PMC4664688

[56] Kanehisa M, Goto S KEGG: kyoto encyclopedia of genes and genomes. Nucleic Acids Res. 2000;28(1):27–30. 10592173 10.1093/nar/28.1.27 PMC102409

[57] Bolton EE, Wang Y, Thiessen PA, et al PubChem: integrated platform of small molecules and biological activities. Annu Rep Comput Chem. 2008;4:217–241, Elsevier

[58] Ørskov E, Oltjen R Influence of carbohydrate and nitrogen sources on the rumen volatile fatty acids and ethanol of cattle fed purified diets. J Nutr. 1967;93(2):222–228. 6069541 10.1093/jn/93.2.222

[59] Spinhirne JP, Koziel JA, Chirase NK Sampling and analysis of volatile organic compounds in bovine breath by solid-phase microextraction and gas chromatography–mass spectrometry. J Chromatogr A. 2004;1025(1):63–69. 14753672 10.1016/j.chroma.2003.08.062

[60] Hamer HM, De Preter V, Windey K, et al Functional analysis of colonic bacterial metabolism: relevant to health?. Am J Physiol Gastrointest Liver Physiol. 2011;302(1):G1–G9. 22016433 10.1152/ajpgi.00048.2011 PMC3345969

[61] Yokoyama MT, Carlson JR Production of skatole and para-cresol by a rumen Lactobacillus sp. Appl Environ Microbiol. 1981;41(1):71–76. 16345702 10.1128/aem.41.1.71-76.1981 PMC243641

[62] Bursell E, Gough A, Beevor P, et al Identification of components of cattle urine attractive to tsetse flies, Glossina spp. (Diptera: glossinidae). Bull Entomol Res. 1988;78(2):281–291.

[63] Martín J, Carranza J, López P, et al A new sexual signal in rutting male red deer: age related chemical scent constituents in the belly black spot. Mamm Biol-Z Säugetierkunde. 2014;79(6):362–368.

[64] Dehnhard M, Bernal-Barragan H, Claus R Rapid and accurate high-performance liquid chromatographic method for the determination of 3-methylindole (skatole) in faeces of various species. J Chromatogr B Biomed Sci Appl. 1991;566(1):101–107. 10.1016/0378-4347(91)80114-r 1885705

[65] Claus R, Raab S. Influences on skatole formation from tryptophan in the pig colon In: G. Heuther, W. Kochen, T. J. Simat, H. Steinhart, editors. Tryptophan, Serotonin, Melatonin. Boston (MA): Springer; 1999 p. 679–684. 10.1007/978-1-4615-4709-9_87 10721118

[66] DeHaan R, Chen Y Multiple connexins and asymmetric currents in embryonic cardiac gap junctions. In: Y. Kanno, Y. Kataoka, Y. Shibata, T. Shimazu, editors. Intercellular Communication through Gap Junctions. Progress in Cell Research, (4). Amsterdam: Elsevier; 1995. p. 187–200.

[67] Aoki KF, Kanehisa M Using the KEGG database resource. Curr Protoc Bioinform. 2005;11(1) 1–12. 10.1002/0471250953.bi0112s11 18428742

[68] Jeon BS, Choi O, Um Y, et al Production of medium-chain carboxylic acids by Megasphaera sp. MH with supplemental electron acceptors. Biotechnol Biofuels. 2016;9(1):129. 27340431 10.1186/s13068-016-0549-3 PMC4918077

[69] Brunet P, Dou L, Cerini C, et al Protein-bound uremic retention solutes. Adv Renal Replace Ther. 2003;10(4):310–320. 10.1053/j.arrt.2003.08.002 14681860

[70] Lee J-H LJ Indole as an intercellular signal in microbial communities. FEMS Microbiol Rev. 2010;34(4):426–444. 20070374 10.1111/j.1574-6976.2009.00204.x

[71] Nelson DL, Cox MM Lehninger Princ Biochem Lect Notebook. 4th ed. New York: W. H. Freeman; 2004.

[72] Mochalski P, King J, Haas M, et al Blood and breath profiles of volatile organic compounds in patients with end-stage renal disease. BMC Nephrol. 2014;15(1):43. 24607025 10.1186/1471-2369-15-43 PMC3984739

[73] Spraker T, O‘Rourke KI, Balachandran A, et al Validation of monoclonal antibody F99/97.6. 1 for immunohistochemical staining of brain and tonsil in mule deer (Odocoileus hemionus) with chronic wasting disease. J Vet Diag Invest. 2002;14(1):3–7. 10.1177/104063870201400102 12680636

[74] Haley NJ, Mathiason CK, Carver S, et al Sensitivity of protein misfolding cyclic amplification versus immunohistochemistry in ante-mortem detection of chronic wasting disease. J General Virol. 2012;93(5):1141–1150. 10.1099/vir.0.039073-0 PMC3541800 22278825

[75] Haley NJ, Siepker C, Walter WD, et al Antemortem detection of chronic wasting disease prions in nasal brush collections and rectal biopsies from white-tailed deer by real time quaking-induced conversion. J Clin Microbiol. 2016;54(4):1108–1116. 10.1128/JCM.02699-15 PMC4809927 26865693

[76] Hibler CP, Wilson KL, Spraker TR, et al Field validation and assessment of an enzyme-linked immunosorbent assay for detecting chronic wasting disease in mule deer (Odocoileus hemionus), white-tailed deer (Odocoileus virginianus), and Rocky Mountain elk (Cervus elaphus nelsoni). J V Diag Invest. 2003;15(4):311–319. 10.1177/104063870301500402 12918810

[77] Seelig DM, Mason GL, Telling GC, et al Chronic wasting disease prion trafficking via the autonomic nervous system. Am J Pathol. 2011;179(3):1319–1328. 21777560 10.1016/j.ajpath.2011.05.057 PMC3157164

[78] Gowda H, Ivanisevic J, Johnson CH, et al Interactive XCMS Online: simplifying advanced metabolomic data processing and subsequent statistical analyses. Anal Chem. 2014;86(14):6931–6939. 24934772 10.1021/ac500734c PMC4215863

[79] Hoover CE, Davenport KA, Henderson DM, et al Pathways of prion spread during early chronic wasting disease in deer. J Virol. 2017;91(10):e00077–17. 28250130 10.1128/JVI.00077-17 PMC5411598

[80] Varmuza K, Filzmoser P Introduction to multivariate statistical analysis in chemometrics. Boca Raton, FL: CRC; 2009.

[81] Atarashi R, Wilham JM, Christensen L, et al Simplified ultrasensitive prion detection by recombinant PrP conversion with shaking. Nat Methods. 2008;5(3):211. 18309304 10.1038/nmeth0308-211

[82] Fletcher RH, Fletcher SW, Fletcher GS Clinical epidemiology: the essentials. Baltimore (MD): Lippincott Williams & Wilkins; 2012.

[83] Park J-H, Choi Y-G, Park S-J, et al Ultra-efficient Amplification of Abnormal Prion Protein by Modified Protein Misfolding Cyclic Amplification with Electric Current. Mol Neurobiol. 2018;55(2):1630–1638. 28194643 10.1007/s12035-017-0431-8 PMC5820375

[84] Danian X, Shilei L Introduction of an Automated Mass Spectral Deconvolution and Identification System─ AMDIS [J]. Mod Sci Instrum. 2002;6:017.

[85] O‘Rourke K, Baszler T, Besser T, et al Preclinical diagnosis of scrapie by immunohistochemistry of third eyelid lymphoid tissue. J Clin Microbiol. 2000;38(9):3254–3259. 10970367 10.1128/jcm.38.9.3254-3259.2000 PMC87369

